# TOPICAL 0.03% ATROPINE VS. 15% ALUMINUM CHLORIDE IN TREATING MULTIPLE ECCRINE HIDROCYSTOMAS: A RANDOMIZED SINGLE BLIND CONTROLLED STUDY

**DOI:** 10.4103/0019-5154.60352

**Published:** 2010

**Authors:** Ehsani Amirhoushang, Mirshams Shashahani Mostafa, Akhyani Maryam, Noormohamadpour Pardis, Noormohamadpour Pedram

**Affiliations:** *From the Department of Dermatology, Tehran University of Medical Sciences, Razi Hospital, Tehran, Iran.*

**Keywords:** *Atropine*, *aluminum chloride*, *multiple eccrine hidrocystomas*

## Abstract

**Background:**

Multiple eccrine hidrocystomas pose a significant treatment challenge due to their facial location and tendency to scar after traditional surgical and other destructive modalities.

**Aims:**

To compare two frequently used non-destructive therapeutic modalities.

**Materials and Methods:**

Thirty patients with multiple eccrine hidrocystomas were enrolled in the study. They used topical 0.03% Atropine cream and 15% AlCl_3_ solution on left and right sides of their face randomly for 4 weeks. All the patients were visited before commencing the therapy as well as two and four weeks later by a dermatologist, blinded to the drugs and the number of lesions.

**Results:**

Twenty nine patients (25 females, four males) completed the study. The mean reduction in the number of lesions was significantly higher with Atropine cream in comparison with AlCl_3_ solution, 10.2±7.4 vs. 6.2±5.3 (*P* < 0.05). There were no recurrences after three months follow-up in both groups.

**Conclusion:**

It seems that both Atropine and AlCl_3_ are useful therapies in eccrine hidrocytomas but the former might be more effective. We think that other randomized clinical trials with larger sample sizes are needed.

## Introduction

Eccrine hidrocystomas are benign cystic lesions which originate from eccrine glands duct[1] predominantly located on the face - eyelid and cheek regions.[2,3]

These lesions are more frequent in females than males.[4] They present as a 1-3 mm translucent yellowish to slightly blue cysts. Eccrine hidrocystoma is currently classified in two subtypes according to the numbers of lesions.[5] Multiple cysts frequently present in periorbital, forehead and malar regions and progress under high temperature and excessive sweating.[3] Treatment of multiple eccrine hidrocystoma lesions is questionable despite the good response of solitary lesion to surgical excision; because of their location and subsequent cosmetic complication.[1]

Therapeutic options include medical and surgical modalities. Surgical excisions, electrocuterry, dermabrasion, topical and oral anticholinergic agents,[1,6,7] botulinum toxin[8] And Pulsed-Dye Laser[9] have been reported as treatment options in these cases.

Despite all these attempts, an agreed-upon treatment for this condition has not yet been formulated. Regarding the eccrine origin of the lesions, the pathogenesis of eccrine hidrocystoma is particularly similar to hyperhydrosis. Hence the first line treatment for hyperhidrosis is 20% (AlCl_3_.6H_2_O) in absolute alcohol.[10] This solution seems to be a reasonable option for the treatment of eccrine hidrocystoma as well.

## Materials and Methods

The study aims to assess the efficacy and safety of 15% (AlCl_3_.6H_2_O) in absolute alcohol solution in treatment of multiple eccrine hidrocistoma compared with topical Atropine 0.03% in cream based on eucerin. In consultation with a pharmacologist assistant, we found that we could not prepare stable solution based Atropine, so we had to use cream based Atropine preparation. Also, to minimize the risk of local irritation, we used 15% (AlCl_3_.6H_2_O) in absolute alcohol solution instead of 20% solution.

The study was designed as a randomized, single blind, controlled trial. All the volunteer patients who referred to our clinic with diagnosis of multiple eccrine hidrocystoma during the summer of 2007, after confirming the diagnosis by three board certified academic dermatologists, were eligible for the study. The study was conducted in accordance with Declaration of Helsinki and approved by the local ethics committees.

Patients were excluded if they had received any treatment for these lesions in the previous year (apart from sunscreens), were pregnant or had any history of sensitivity to drugs used in the study. Diagnosis of eccrine hidrocystoma was confirmed through clinical examination by three Board certified academic dermatologists based on clinical grounds such as multiplicity of lesions, recurrent nature of them and weeping fluid when pricked by needle, and number of lesions on each side of face was recorded in special questionnaires.

During the study period, no other therapeutics was used. After informed consent, 30 patients were enrolled in this study. All patients had multiple eccrine hidrocystoma lesions on both sides of their face. For each patient, a questionnaire was completed and appropriate method of solution and cream application was actually demonstrated. For each patient, 15% AlCl_3_ in absolute alcohol and Atropine containing cream randomly prescribed for different sides of face lesions by a participating dermatologist as manager of research. Hence, some patients received solution on right side lesions and cream on left side lesions and vice versa for others. Patients were advised against use of Atropine containing cream very near the eyelids (a distance of at least 0.5 cm should be considered). This information stored secure for each patient and participating examiner dermatologist was blinded to this information. Patients visited at second and fourth weeks after beginning the study and were examined by the dermatologist; any changes in number of lesions were documented in special questionnaires designed for this purpose. One patient was excluded from study after he discontinued his treatment regimen.

Patients applied solution and cream to absolutely dry skin at bed time, 30 to 60 minutes after cleansing the face with water; they washed the face with water the following morning. If there were any signs of irritation or other sensitivity to any drug used, the therapy was discontinued.

### Statistical analysis

Data analysis was performed using SPSS for windows (v 13.0) statistical package. *t*-test used for comparisons. The differences were statistically significant for *P* < 0.05.

### Side effects

Dryness of skin (30%) and local skin irritation (7%) were significant adverse effects of cream containing 0.03% Atropine, and we had no ophthalmic side effect. Adverse effects in the 15% (AlCl_3_.6H_2_O) group were burning sensation and local irritation (22%), erythema (10%), and dryness and skin (25%). One patient discontinued the therapy following severe irritation caused by the solution. All of her skin irritation cleared with a week's use of topical triamcinolone.

## Results

Twenty nine patients, 25 (86.2%) female and four (13.8%) male completed the study. The age range of patients was between 29 to 61 years, with the mean age of 40.5±8.74 and 43.6±7.59 years for men and women, respectively.

Before commencing the therapy, mean number of lesions on each side of face was 20.5±13.4 for left side and 20.7±12.9 for right side of the face (*P* > 0.1, not significant). After four weeks of therapy, mean reduction of lesions was 10.2±7.4 and 6.2±5.3 for Atropine cream and (AlCl_3_.6H_2_O) in absolute alcohol solution, respectively (*P* < 0.05).

This means that mean reduction of lesion for 0.03% Atropine cream is significantly better than traditionally accepted therapy with (AlCl_3_.6H_2_O) in absolute alcohol solution, and reduction rate is about 50%. There were no recurrences after three months follow-up in both groups.

## Discussion

AlCl_3_.6H_2_O produces Aluminum ion and hydrochloric acid in the presence of water. The produced acid can cause skin irritation and damage.[11] Therefore, it is imperative to apply this solution to absolutely dry skin, never in less than 30 minutes to two hours after washing. Also the solution should not be used on irritated, wet, ulcerated, or recently shaved skin. It is recommended to apply the solution at bed time, and wash the skin the following morning.[10,12,13]

The antiperspirant mechanism of AlCl_3_.6H_2_O is by physical blockage of sweat gland ducts and vacuolization and atrophy of secretary cells. It penetrates to terminal end of eccrine ducts in epidermis by reflux entrance and causes shrinkage of the ducts.[10,12]

Atropine 1% ointment or solution in case reports has been used in eccrine hidrocystoma management successfully but several anti cholinergic side effects such as blurred vision or mouth dryness were seen after this therapeutic option.[7] Because of these adverse effects, we used a lower concentration of atropine (0.03%) and as said earlier, we had no ocular side effects or dryness of oral mucosa. At least in one study topical atropine ointment was unsuccessful in treating multiple eccrine hidrocystomas.[14]

Surgical excision of individual lesions is effective but limited by the risk of scarring and ectropion formation.[15,16] Lesional drainage and cautery are other therapeutic modalities in eccrine hidrocystomas but frequently; recurrence of lesions after three to six weeks were noted[17] although in the present study we had no recurrences after three months of follow-up in both the enrolled groups.

A 585-nm flashlamp-pumped pulse dye laser (PDL) leads to satisfactory improvement in eccrine hidrocystomas, without recurrence 18 months after.[18] There are reports questioning the effect of PDL on multiple eccrine hidrocystoma.[19]

In comparison to these therapeutic modalities, 0.03% Atropine cream and, 15% AlCl_3_.6H_2_O are not consuming and low cost without any significant adverse effects. In conclusion, it seems that both these therapies are effective in eccrine hidrocystomas treatement and 0.03% Atropine cream is significantly better than 15% AlCl_3_.6H_2_O, although we think other randomized clinical trials with larger sample sizes are needed.
